# Prevalence and factors associated with hyperphosphatemia in continuous ambulatory peritoneal dialysis patients: A cross-sectional study

**DOI:** 10.3389/fmed.2023.1142013

**Published:** 2023-04-14

**Authors:** Xiaojing Yin, Fan Zhang, Yan Shi

**Affiliations:** ^1^Tongji University School of Medicine, Shanghai, China; ^2^Department of Nephrology, Longhua Hospital Shanghai University of Traditional Chinese Medicine, Shanghai, China

**Keywords:** continuous ambulatory peritoneal dialysis, hyperphosphatemia, prevalence, factors, cross-sectional study

## Abstract

**Background:**

Hyperphosphatemia remains a major complication in patients with Continuous ambulatory peritoneal dialysis (CAPD) leading to increased morbidity and mortality. However, phosphorus management still has many challenges.

**Objective:**

This study aimed to investigate the prevalence and factors of hyperphosphatemia among continuous ambulatory peritoneal dialysis patients in a tertiary public hospital in Shanghai, China.

**Methods:**

The single-center cross-sectional study recruited end-stage renal failure patients who received continuous ambulatory peritoneal dialysis (CAPD) for at least 3 months. The participants aged 18–80 years had undergone CAPD between 1 July 2021 and 30 May 2022, in Shanghai, China.

The patients’ sociodemographic, clinical, and laboratory data were collected prospectively from medical records and *via* face-to-face interviews. A sample size of convenience decides the sample size. This study used the information-motivation-behavioral (IMB) skills model as a theoretical framework. The questionnaire included knowledge and behavior of diet and medication in patients with hyperphosphatemia of chronic kidney disease, self-efficacy for managing chronic disease, and social support rating scale. Univariate analysis and binary logistic regression were performed to identify the influencing factors of hyperphosphatemia by SPPS 27.0.

**Results:**

In total, 141 CAPD patients (73% hyperphosphatemia) were included in the final analysis. In logistic regression analysis, dialysis vintage (OR: 0.975, 95%CI: 0.957–0.993), dialysis exchanges (OR: 0.317, 95%CI: 0.131–0.768), urine output (OR: 0.997, 95%CI: 0.995–0.999), serum albumin (OR: 1.166, 95%CI:1.008–1.349), serum creatinine (OR: 1.005, 95%CI: 1.001–1.008), hyperphosphatemia knowledge behavior score (OR: 0.888, 95%CI: 0.797–0.991), and social support level (OR: 0.841, 95%CI:0.765–0.925) were the influencing factors of hyperphosphatemia.

**Conclusion:**

Hyperphosphatemia is a frequent complication in CAPD patients. Dialysis vintage, dialysis exchanges, urine output, serum albumin, serum creatinine, hyperphosphatemia knowledge behavior, and social support were the associated factors of hyperphosphatemia in CAPD patients. It is crucial for healthcare providers to maintain phosphorus balance among CAPD patients using phosphorus management strategies.

## 1. Introduction

Chronic kidney disease (CKD) is defined as renal structural or functional abnormalities for 3 months, with a prevalence of 13.4% worldwide (1). End-stage renal disease (ESRD) was defined as a need for renal replacement therapy, CKD stage G5 (estimated glomerular filtration rate [eGFR] ≤ 15 mL/min per 1.73 m^2^) (2). Peritoneal dialysis (PD) is an effective strategy of renal replacement therapy for patients with ESRD, characterized by multiple advantages including the protection of residual renal function, hemodynamic stability, home care and be more independent, and patients have a higher quality of life and survival. With the development of PD technology and the support of national policies, it has been widely used and promoted clinically in remote areas (3). About 10–20% of ESRD patients worldwide are treated with PD.

Phosphorus is mainly excreted through the kidneys. The target serum phosphorus concentrations of ≥1.13 mmol/L and ≤ 1.78 mmol/L were based on the target range from the Kidney Disease Improving Global Outcomes (KDIGO) guidelines. In CAPD patients, hyperphosphatemia is defined as serum phosphorus >1.78 mmol/L (4). A cross-sectional analysis of the China Dialysis Calcification Study (CDCS) showed that poor compliance to diet, phosphorus binders, and dialysis treatment in Chinese PD patients was 65.7% (5), which is much higher than that in developed countries (6). Long-term hyperphosphatemia can lead to symptoms such as pruritus, convulsions, limb ulceration, fracture bone pain, vascular and soft tissue calcification, and hyperparathyroidism, which seriously threatens the survival health and life quality of patients (7). Thus, nephrologists should be encouraged to treat and prevent the progression of hyperphosphatemia in CAPD patients.

The information-motivation-behavioral (IMB) skills model, proposed by Fisher (8) and Fisher to explain human immunodeficiency virus (HIV)-related behaviors, has been widely used to indicate positive changes in health behaviors in various populations. The IMB model proposes a pre-survey of the study population before the intervention to understand and collect data and information to assess the main factors influencing behavior change before the intervention can be implemented. Previous research has shown that the IMB model is an appropriate and applicable model to explain and predict the self-care behavior of Chinese PD patients (9). Our study members found that patients’ knowledge, self-efficacy, and serum phosphorus management compliance may be potential factors affecting serum phosphorus. Therefore, this study aimed to use the IMB model to guide and assess the characteristics associated with hyperphosphatemia in CAPD individuals. A knowledge behavior questionnaire investigated the information behavior part of this study. The motivation level was divided into self-motivation and social motivation levels, which were investigated by self-efficacy and social support scales.

## 2. Methods

### 2.1. Study design

This was a single-center cross-sectional survey study. We recruited 141 end-stage renal failure who received continuous ambulatory peritoneal dialysis (CAPD) for at least 3 months at a tertiary public hospital peritoneal dialysis center in Shanghai between 1 July 2021 and 30 May 2022. This study was ethically approved by the Medical Ethics Committee of Longhua Hospital, Shanghai University of Traditional Chinese Medicine, on 27 May 2020 (reference number: 2021LCSY071). All individuals gave their prior consent before placing any study.

### 2.2. Sample size calculation

We used Xiao Shunzhen’s (10) rough estimation sample size method. The sample size was 5–10 times the number of the highest items in the questionnaire. The equation for calculating the number of participants is the following: Sample size = [Max (number of entries)* (5–10)] * [1+ (10–30%)]. In our study, the highest items of the questionnaire is a questionnaire on knowledge and behavior related to diet and medication taking in patients with chronic kidney disease hyperphosphatemia, with 16 items. According to the sample size calculation equation, the sample size = [16 * (5–10)] * [1 + (10–30%)] that is 88–208. Considering that there may be 20% of invalid questionnaires, the sample size of this study was between 110 and 260. Based on the actual number of clinical peritoneal dialysis patients, a final sample size of 141 cases was calculated.

### 2.3. Inclusion and exclusion criteria

We recruited end-stage renal failure who received regular, continuous ambulatory peritoneal dialysis (CAPD) for at least 3 months between 1 July 2021 and 30 May 2022, in Shanghai, China. Inclusion criteria were: ① age 18–80 years, ② had completed theoretical and operational training about diet and medication, ③ be able to read and communicate, and ④ voluntary participation in this study. Those who could not cooperate with the investigation, such as functionally impaired, cognitive dysfunction, and severe physical disease, were excluded. All participants used the conventional PD solutions (Dianeal 1.5, 2.5% dextrose; Baxter Healthcare, Guangzhou, China). Y sets and twin bag systems were used in all participants. The study conformed with the principles in the Declaration of Helsinki, and all included patients signed an informed consent form before the investigation.

### 2.4. Definitions

Kidney Disease Improving Global Outcomes defined hyperphosphatemia as serum phosphorus >1.78 mmol/L in peritoneal dialysis patients.

### 2.5. Data collection

The sociodemographic data, designed by literature analysis and consultation with experts consistent with the purpose of the study, including gender, age, education, marital status, occupation, and economic status, were collected in face-to-face interviews.

The clinical and laboratory data were collected from medical records, including the patient’s primary cause of peritoneal dialysis, dialysis vintage, dialysis exchanges, urine output, ultrafiltration volume, and the type of phosphorus binders taken when we investigated. After admission, laboratory parameters were collected first for fasting blood, including serum phosphorus, calcium, parathyroid hormone, albumin, and creatinine.

Continuous ambulatory peritoneal dialysis patients’ knowledge and behavior related to diet and medication of phosphorus management were measured by a questionnaire on knowledge and behavior related to diet and medication taking in patients with chronic kidney disease hyperphosphatemia developed by Yuexian Shi et al. (11). The questionnaire included 17 items, including four dimensions covering disease-related knowledge of dietary, medication-taking, and hyperphosphatemia-related behavior. The 17th item is an open-ended question without scoring. The total score of the questionnaire was 0–36; the higher the score, the better the patient’s knowledge and phosphorus management compliance. The Cronbach’s α coefficient of each dimension of the questionnaire ranged from 0.77 to 0.90, and the retest reliability coefficient ranged from 0.74 to 0.93, which had good internal consistency.

Self-efficacy evaluated patients’ confidence in phosphorus management for managing chronic disease developed by Lorig et al. (12), which has been widely used in chronic diseases. The higher the score, the higher the level of self-efficacy and the higher the level of motivation and confidence in performing self-management behaviors. The Cronbach’s alpha coefficient of this scale is 0.96, which has high reliability and validity.

We used the social support rating scale to assess the level of social support of CAPD patients, which was developed by Shuiyuan Xiao (13) and has been widely used in clinical studies and contains three dimensions: objective support, emotional support, and utilization of social support. A total score of ≤22 indicates low-level social support, 23–44 shows medium-level social support, and 45–66 indicates high-level social support. The Cronbach’s coefficient for this scale was 0.633–0.896.

### 2.6. Statistical analysis

One hundred and forty-one questionnaires of the 147 distributed initially were returned, resulting in a response rate of 95.92%. Four invalid questionnaires were due to the lack of relevant laboratory indicators and two were completed in less than 1 min. Statistical Package for Social Sciences (SPSS) version 27.0 for windows was used to analyze the data. Data were analyzed with the Statistical Package for the Social Sciences (SPSS) version 27.0. Continuous variables were reported as mean and standard deviation (SD) for normal distribution or median (interquartile range [IQR]) for skewed distribution and categorical variables as frequencies (percentages). Student’s t-test or one-way analysis was used for univariate analysis of hyperphosphatemia between study variables. A multivariable logistic regression analysis was carried out to identify factors associated with hyperphosphatemia, and variables with *p* < 0.05 were considered statistically significant.

## 3. Results

The average age of all study participants was 57 years old (mean: 57, standard deviation: 13.0), with 39% male subjects. The primary causes of PD were diabetic nephropathy (*n* = 35, 24.8%), hypertensive nephropathy (*n* = 65, 46.1%), and chronic glomerulonephritis (*n* = 22, 15.6%). Most patients (62.4%) had used lanthanum carbonate phosphorus binder. Compared with people without hyperphosphatemia, it showed no differences in the general demographic characteristics (Table 1).

**Table 1:** Demographic characteristics and clinical characteristics of the study population.

Variables		Total	Hyperphosphatemia		*p-*value
		(*n* = 141)	no (*n* = 38)	yes (*n* = 103)	
Gender, *n* (%)	Male	55 (39)	15 (27.3)	40 (72.7)	0.945^a^
	Female	86 (61)	23 (26.7)	63 (73.3)	
Age (years), mean ± SD		56.60 ± 12.96	57.95 ± 13.78	56.11 ± 12.67	0.456^b^
BMI (kg/m^2^), mean ± SD		22.29 ± 3.02	22.02 ± 3.22	22.39 ± 2.95	0.475^b^
Marital Status, *n* (%)	Unmarried	15 (10.6)	6 (40.0)	9 (60.0)	0.228^a^
	Married	126 (89.4)	32 (25.4)	94 (74.6)	
Education level, *n* (%)	Primary school and Below	14 (9.9)	7 (18.4)	7 (6.8)	0.156^a^
	Junior middle school	40(28.4)	10 (26.3)	30 (29.1)	
	High school or junior college	49(34.8)	9 (23.7)	40 (38.8)	
	College	19 (13.5)	7 (18.4)	12 (11.7)	
	Bachelor’s degree or above	19 (13.5)	5 (13.2)	14 (13.6)	
Occupational, *n* (%)	On-the-job	18 (12.8)	1 (2.6)	17 (16.5)	0.145^a^
	Liberal professions	14 (9.9)	3 (7.9)	11 (10.7)	
	Retirement	67 (47.5)	21 (55.3)	46 (44.7)	
	Unemployed	42 (29.8)	13 (34.2)	29 (28.2)	
Financial burden, *n* (%)	None	33 (23.4)	5 (13.2)	28 (27.2)	0.172^a^
	Light	64 (45.4)	18 (47.4)	46 (44.7)	
	Heavy	44 (31.2)	15 (39.5)	29 (28.2)	
The primary cause of PD, *n* (%)	Diabetic nephropathy	35 (24.8)	12 (31.6)	23 (22.3)	0.546^a^
	Hypertension	65 (46.1)	14 (36.8)	51 (49.5)	
	Chronic glomerulonephritis	22 (15.6)	6 (15.8)	16 (15.5)	
	Others	19 (13.5)	6 (15.8)	13 (12.6)	0.546^a^
Taking phosphorus binders, *n* (%)	None	10 (7.1)	9 (90.0)	1 (10.0)	<0.001^a^
	Lanthanum carbonate	88 (62.4)	18 (20.5)	70 (79.5)	
	Calcium acetate	31 (22.0)	11 (35.5)	20 (64.5)	<0.001^a^
	Calcium carbonate	3 (2.1)	0 (0)	3 (100)	
	Others	9 (6.4)	0 (0)	9 (100)	

^a^*p*-value of the chi-square test for independence; ^b^*p*-value of *t*-test for independent samples; BMI, body mass index; PD, peritoneal dialysis.

The dialysis vintage was 29 (10, 55), and the dialysis vintage in patients with hyperphosphatemia was shorter relative to that in patients without hyperphosphatemia [40 (13, 92.75) vs. 23 (10, 52), *p* = 0.035]. Univariate analysis of daily dialysis exchanges, ultrafiltration volume, and urine output was not statistically different (Table 2).

**Table 2:** Clinically dialysis-relevant data of the study population.

Variables	Total	Hyperphosphatemia		*P*-value
	(*n* = 141)	no (*n* = 38)	yes (*n* = 103)	
Dialysis vintage (months)	29 (10, 55)	40 (13, 92.75)	23 (10, 52)	0.035
Dialysis exchanges(times/day)	4 (4, 5)	4 (3.75, 5)	4 (4, 4)	0.294
Urine output(mL/day)	460 (65, 800)	500 (0, 1,000)	400 (80, 800)	0.309
Ultrafiltration volume(mL/day)	360 (200, 775)	375 (200, 662.5)	360 (200, 800)	0.404

Data are expressed as median (interquartile range).

Serum phosphorus (P), serum calcium (Ca), serum parathyroid hormone (PTH), serum albumin (Alb), and serum creatinine (Scr) were measured and analyzed. The prevalence of hyperphosphatemia was 73% (103/141). It showed statistically significant differences in the PTH and Scr (*p* < 0.05) between hyperphosphatemia and non-hyperphosphatemia CAPD patients (Table 3).

**Table 3:** Biochemical parameters of the study population.

Variables	Total	Hyperphosphatemia		*P*-value
	(*n* = 141)	no (*n* = 38)	yes (*n* = 103)	
Serum Ca (mmlo/L)	2.23 (2.095, 2.34)	2.23 (2.11, 2.30)	2.23 (2.09, 2.35)	0.759
Serum PTH (pg/mL)	281.3 (148, 517)	196.5 (130.65, 383)	300 (152, 602.4)	0.031
Serum Alb (g/dL)	32.8 (29.35,36.9)	30.7 (28.9, 34.83)	32.9 (29.8, 37.4)	0.077
Scr (mmlo/L)	832.1 (675, 1,000)	725 (590, 906.75)	882.3 (700, 1,016)	0.005

Data are expressed as median (interquartile range).

The total score of the knowledge and behavior questionnaire for hyperphosphatemia in this study was (21.47 ± 5.471), with a higher level of knowledge and behavior in PD patients without hyperphosphatemia [(21.47 ± 5.471) vs. (18.71 ± 4.926), *p* = 0.005]; the total score of the chronic disease self-efficacy scale was (41.92 ± 7.231), the level of self-efficacy was relatively low in patients with hyperphosphatemia; the total score of social support scale was (41.97 ± 7.172), the level of social support was higher in PD patients without hyperphosphatemia (Table 4).

**Table 4:** Questionnaire score of the study population.

Variables	Total	Hyperphosphatemia		*p-*value
	(*n* = 141)	no (*n* = 38)	yes (*n* = 103)	
Hyperphosphatemia dietary and medication knowledge and behavior	19.45 ± 5.21	21.47 ± 5.47	18.71 ± 4.93	0.005
Self-efficacy	38.13 ± 9.85	41.92 ± 7.23	36.74 ± 10.33	0.005
Social support	37.40 ± 7.56	41.97 ± 7.17	35.72 ± 7.00	<0.001

Data are expressed as the mean ± standard deviation.

Multifactorial logistic regression analysis was applied to determine whether hyperphosphatemia was the dependent variable (no occurrence = 0, event = 1) and variables with univariate analysis (*p* < 0.05), and variables that may influence hyperphosphatemia obtained from clinical experts’ recommendations and clinical experience were used as independent variables for multifactorial logistic regression analysis that showed dialysis vintage, dialysis exchanges, urine output, serum Alb, Scr, level of hyperphosphatemia knowledge behavior, and level of social support were influential factors in the development of hyperphosphatemia (Table 5).

**Table 5:** Logistic regression analysis of the factors influencing hyperphosphatemia of the study population.

Variables	*B*	SE	*p*-value	OR	OR95%CI
Age (year)	−0.025	0.024	0.303	0.976	0.931–1.023
BMI (kg/m^2^)	−0.021	0.096	0.827	0.979	0.812–1.181
Dialysis vintage (months)	−0.026	0.009	0.006	0.975	0.957–0.993
Dialysis exchanges (times/day)	−1.149	0.452	0.011	0.317	0.131–0.768
Urine output (mL/day)	−0.003	0.001	0.001	0.997	0.995–0.999
Ultrafiltration volume (mL/day)	0	0.001	0.89	1	0.998–1.002
Serum Ca (mmlo/L)	−0.457	1.341	0.733	0.633	0.046–8.767
Serum PTH (pg/mL)	0.001	0.001	0.264	1.001	0.999–1.003
Serum Alb (g/dL)	0.154	0.074	0.039	1.166	1.008–1.349
SCR (mmlo/L)	0.004	0.002	0.006	1.005	1.001–1.008
Hyperphosphatemia dietary and medication knowledge and behavior	−0.118	0.056	0.033	0.888	0.797–0.991
Self-efficacy	−0.044	0.031	0.158	0.957	0.9–1.017
Social support	−0.173	0.048	<0.001	0.841	0.765–0.925

Hosmer–Lemesho, H–L test: *p* = 0.869 > 0.05.

## 4. Discussion

Hyperphosphatemia in dialysis patients is as high as 40% (14). Our study showed that the prevalence of hyperphosphatemia among 141 CAPD patients was 73%, significantly higher than in previous studies. In patients with poorly controlled hyperphosphatemia over time, taking declining peritoneal function and reducing or no renal function remains into account; patients may have to be transferred to hemodialysis.

Serum creatinine (Scr) is measured as a standard indicator of renal function. A higher Scr level indicates more severe renal impairment, also a muscle mass marker. In our study, Scr stayed significantly associated with hyperphosphatemia after adjusting for residual diuresis and remained an independent risk factor, consistent with the findings of the Malgorzata Debowska study (15). Higher muscle mass frequently correlates with higher nutritional such as fish, meat, eggs, and milk intake, also reflected by more elevated serum Alb in patients with hyperphosphatemia, which is just short of statistical significance in our study (OR: 1.166, 95%CI:1.01–1.35, *p* = 0.039). Hyperphosphatemia is a central problem in chronic kidney disease-mineral and bone disorder (CKD-MBD); serum P, Ca, and serum PTH levels interact. Our study also showed that as serum Ca levels increased, serum P decreased and elevated PTH levels contributed to the increase in serum p, consistent with previous research (16). Elevated phosphorus levels are closely related to and interact with low serum Ca and high PTH levels. Serum P and Serum Ca are recommended to be measured every 1–3 months in CAPD patients. The PTH is measured every 3–6 months (17). Timely monitoring, early detection, and early intervention of problems are essential for phosphorus control.

The age in this study was 55 (46, 65), with 72.8% being middle-aged and elderly patients. We found reduced phosphorus with aging, which is inconsistent with the results of previous studies (18). The incidence of malnutrition in peritoneal dialysis patients ranges from 18 to 75%, with a higher incidence of malnutrition in older patients than in young adults (19). Serum phosphorus levels that decrease with aging could signify sarcopenia and malnutrition. The prevalence of hypophosphatemia in the malnourished population was (10.4%) (20), and poor muscle contraction is one symptom of hypophosphatemia. Both hyperphosphatemia and hypophosphatemia can occur in CAPD patients, and malnutrition may be an influential factor in the imbalance of serum phosphorus. However, the mechanism of hyperphosphatemia is not entirely understood and occurs in PD patients; there are still many challenges in serum P control (21). Dietary management is essential to increase serum Alb levels to improve malnutrition. Up-to-date clinical decision-making (17) suggests limiting dietary phosphorus intake (900 mg/d) in treating hypophosphatemia in CKD, avoiding unnecessary nutritional phosphorus intake, and maintaining a high biological value source of protein. The Chinese clinical practice guidelines for the nutritional treatment of chronic kidney disease (2021 edition) recommend limiting phosphorus intake without restricting protein intake and choosing foods with a low phosphorus/protein ratio of <12 mg/g (1B) (22). Steaming can be used to remove phosphorus from food and focus on the phosphorus bioavailability of food. It has been shown that low phosphorus dietary intervention >4 months is associated with a mean reduction in a serum phosphate concentration of 0.34 mmol/L (23). Clinical self-management of diet in CAPD patients should prevent both the problem of malnutrition due to excessive phosphorus control and the exacerbation of hyperphosphatemia by increased phosphorus intake due to excessive protein intake.

The decreased glomerular filtration rate is essential for reducing or eliminating urine output in PD patients. Our study also showed that the risk of hyperphosphatemia decreased with increasing daily urine output (OR: 0.997, 95% CI: 0.995–0.999, *p* = 0.001), which is consistent with the results of a previous study (24). Usually, the residual renal function decreases over time, and patients with longer dialysis vintage should have a lower residual renal function and, thus, more hyperphosphatemia. In our study, the age of dialysis vintage was 29 (10, 55) months. In univariate analysis, the dialysis vintage was shorter in patients with hyperphosphatemia. Multifactorial logistic regression analysis showed that the longer the dialysis vintage, the findings were inconsistent with previous studies. We combined the specific peritoneal dialysis patient management situation in our study center to analyze. The peritoneal dialysis center in our study follows up and educates PD patients every 1–3 months in the hospital, outpatient, or by telephone or WeChat. Professional doctors and nurses promptly answer PD patients’ questions in their daily self-management. We also established a WeChat public account to release knowledge about the self-management of PD. As the number of follow-up visits increased with the dialysis time of PD patients, the various forms of health education promoted the increase of PD self-management knowledge and problem-solving ability, which may be the reason for better control of blood phosphorus levels in PD patients with high dialysis vintage. Recommendations for our practice include providing more medical support to peritoneal dialysis patients and improving the self-management of peritoneal dialysis patients, which is essential for serum phosphorus control.

Our study also showed that the risk of hyperphosphatemia was lower with more daily dialysis exchanges (OR: 0.317, 95% CI:0.131–0.768, *p* = 0.011), which is consistent with the findings of previous studies (25). When developing dialysis protocols for patients with PD, the International Society for Peritoneal Dialysis (ISPD) proposed the establishment of high-quality goal-directed peritoneal dialysis (26), which should maintain dialysis adequacy that peritoneal weekly dialysis dose (Kt/V) ≥ 1.7 with minimal dialysis load, provide the best health prognosis for dialysis patients, and maintain stable clinical status. KDIGO guidelines also recommend intensive health education on maintaining dialysis adequacy to better control blood phosphorus levels (27).

When a low phosphorus diet is performed but hyperphosphatemia persists, phosphorus binders should be used, and calcium-free phosphorus binders are recommended (Grade 2B). The types of phosphorus binders investigated in our study were divided into calcium-containing phosphorus binders, such as calcium acetate and calcium carbonate. Non-calcium-containing phosphorus binders such as lanthanum carbonate all have better control of blood phosphorus, with 62.4% of PD patients taking lanthanum carbonate drugs and 10 patients not using phosphorus binder, including one with hyperphosphatemia. The overall situation of phosphorus binders use was good. The correct use of phosphorus binders is essential in phosphorus control and reducing all-cause mortality in patients with PD (28). It has been shown that the non-adherence rate of phosphorus binders taken in ESRD patients is 22–74% (29), and lack of medication Knowledge, high financial burden, low level of family social support, and forgetfulness of missed doses are factors influencing low adherence to phosphorus binders management (30). In our study, the overall hyperphosphatemia diet drug knowledge and behavior level was low at 19.45 ± 5.206, and non-hyperphosphatemia patients had a higher level. Higher levels also showed lower hyperphosphatemia risk (OR: 0.888, 95% CI: 0.797–0.991, *p* = 0.033). Previous studies have shown that health education improves knowledge and behavior of hyperphosphatemia in PD patients to lower serum phosphorus levels (31, 32). Baker T et al.’s research showed that in managing CKD-MBD, patients want more information about diet, phosphorus binders, clinically relevant biochemical abnormalities, and the dangers of uncontrolled hyperphosphatemia (33). However, the current clinical health education approach is mainly passive patient indoctrination, which has limited phosphorus control. Further research on the health education approach to phosphorus management is worthy of further development.

It showed that the risk of hyperphosphatemia decreased as social support increased (OR: 0.841, 95%CI: 0.765–0.925, *p* < 0.001). Previous studies have also shown that social support from healthcare providers and family members is essential in managing phosphorus in PD patients. On the one hand, it is difficult for PD patients to adhere to a low phosphorus diet due to cognitive and dietary habits. Food cooking methods are essential to reduce phosphorus intake, which requires phosphorus management knowledge provided by healthcare professionals and supervision and support from family members. On the other hand, to some extent, the usage of phosphorus binders can increase the financial burden of PD patients. In our study, only 12.77% (18/141) of PD patients worked and most lacked financial resources. The price of phosphorus binders with good efficacy and few side effects was relatively high. Some patients’ low medical reimbursement ratio was a barrier factor for phosphorus binders to use. In this situation, better family economic conditions had somewhat higher affordability of phosphorus binders, which may better phosphorus control in PD patients. In addition to the efficacy and toxicity of phosphorus binders, cheaper and other available drugs should be considered.

## 5. Conclusion

Hyperphosphatemia is a frequent complication in CAPD patients. Dialysis vintage, dialysis exchanges, urine output, serum albumin, serum creatinine, hyperphosphatemia knowledge behavior level, and social support level were the associated factors of hyperphosphatemia in CAPD patients. It is crucial for healthcare providers to provide exceptional care of phosphorus management strategies to keep the phosphorus balance among CAPD patients. At the same time, it is without any doubt that family members or caregivers should be encouraged to participate in the patient’s hyperphosphatemia management.

## 6. Limitations

The limitations of our study are mainly reflected in the following aspects. First, our study recommended a target value of 1.13–1.78 mmol/L for serum P in CAPD patients according to KDIGO, and serum *p* > 1.78 mmol/L was determined as hyperphosphatemia, which is higher than the serum P level for clinical diagnosis of hyperphosphatemia, and the actual prevalence of clinical hyperphosphatemia might be higher. Therefore, the results of the statistical analysis may be somewhat biased when interpreting and applying them to clinical situations. Second, records of residual renal function and PD clearance (weekly Kt/V) and peritoneal equilibrium test were missing from many patients’ cases and unavailable through consultation with patients when we investigated, so they were not included after discussion among our study team. Therefore, in future, such studies can be done with extended follow-up to ensure complete data and, ultimately, more comprehensive results and conclusions. Third, it was a single-center cross-sectional study, and convenient sampling was adopted in our research. The sample size was relatively small and unrepresentative, thus, the findings only represent PD patients at our center and may have some bias in interpretation. Therefore, the findings need to be further confirmed by a multicenter study with a larger sample size.

## Data availability statement

The original contributions presented in the study are included in the article/supplementary material, further inquiries can be directed to the corresponding author.

## Ethics statement

The studies involving human participants were reviewed and approved by the Ethics Committee of Longhua Hospital, Shanghai University of Traditional Chinese Medicine (approval number: 2021LCSY071). The patients/participants provided their written informed consent to participate in this study. Written informed consent was obtained from the individual(s) for the publication of any potentially identifiable images or data included in this article.

## Author contributions

XY designed the study, collected the data, analyzed the data, and drafted the manuscript. FZ drafted and revised the manuscript. YS designed the study and approved the final version of the manuscript.

## Funding

This study was supported by the Longhua Hospital Shanghai University of Traditional Chinese Medicine (grant number: RC-2021-03-07). The funding entity had no role in the study design, data collection, analysis, or publication decision.

## Conflict of interest

The authors declare that the research was conducted in the absence of any commercial or financial relationships that could be construed as a potential conflict of interest.

## Publisher’s note

All claims expressed in this article are solely those of the authors and do not necessarily represent those of their affiliated organizations, or those of the publisher, the editors and the reviewers. Any product that may be evaluated in this article, or claim that may be made by its manufacturer, is not guaranteed or endorsed by the publisher.
