# Corrigendum: Prevalence and factors associated with hyperphosphatemia in continuous ambulatory peritoneal dialysis patients: a cross-sectional study

**DOI:** 10.3389/fmed.2023.1217890

**Published:** 2023-06-29

**Authors:** Xiaojing Yin, Fan Zhang, Yan Shi

**Affiliations:** ^1^Tongji University School of Medicine, Shanghai, China; ^2^Department of Nephrology, Longhua Hospital Shanghai University of Traditional Chinese Medicine, Shanghai, China

**Keywords:** continuous ambulatory peritoneal dialysis, hyperphosphatemia, prevalence, factors, cross-sectional study

In the published article, there was an error. We incorrectly rephrased the sample size calculation equation. A correction has been made to **2. Methods**, “*2.2. Sample size calculation*.” This sentence previously stated:

“According to the sample size calculation equation, the sample size = [16 ^*^ (5–0)] ^*^ [1 + (10–3%)] that is 88–208.”

The corrected sentence appears below:

“According to the sample size calculation equation, the sample size = [16 ^*^ (5–10)] ^*^ [1 + (10–30%)] that is 88–208.”

In the published article, there was an error. We mistakenly stated the author contributions of the paper. A correction has been made to **Author contributions**. This sentence previously stated:

“XY designed the study, collected the data, analyzed the data, and drafted the manuscript. FZ drafted and revised the manuscript. Fenrong Chen, Liuyan Huang, and Huachun Zhang revised the manuscript. YS designed the study and approved the final version of the manuscript.”

The corrected sentence appears below:

“XY designed the study, collected the data, analyzed the data, and drafted the manuscript. FZ drafted and revised the manuscript. YS designed the study and approved the final version of the manuscript.”

In the published article, an author name was incorrectly written as “Fang Zhang.” The correct spelling is “Fan Zhang.”

The authors apologize for these errors and state that these do not change the scientific conclusions of the article in any way. The original article has been updated.

